# We Need a Taxonomy of Working Memory

**DOI:** 10.5334/joc.71

**Published:** 2019-08-08

**Authors:** Anastasia Kiyonaga

**Affiliations:** 1 Helen Wills Neuroscience Institute, University of California, Berkeley, CA, US

**Keywords:** Working memory, Attention, Cognitive Control

## Abstract

A vast and diverse literature describes the relationship between working memory and attention. That literature encompasses tests of the interactions between the two functions, comparisons of the processes underlying them, and theoretical formulations of the cognitive constructs. In a recent review, Oberauer (2019) reins in this varied work to create a roadmap for future research. Here, I delineate several additional considerations to guide the evaluation and development of research into working memory and attention. Namely, working memory is a complex construct that can entail many processes and take on several meanings. Research and theory about working memory—and its relation to attention—must consider what particular demands are being tapped, what type of information is being operated on, and what goal is ultimately at stake.

After recent years of widespread agreement that working memory (WM) and attention are related functions, Oberauer (2019) takes on the formidable task of organizing and evaluating the evidence for this relationship. This review comes at an opportune time, as new work has highlighted the separability of WM and attention, and cautions against using the two interchangeably (Bae & Luck, 2018; Harrison & Bays, 2018; Mendoza-Halliday & Martinez-Trujillo, 2017). Indeed, WM is defined by an obvious distinction from outwardly-oriented (i.e. ‘perceptual’) attention: WM content must be endogenously activated without current sensory input to evoke the representation. Therefore, to treat them as if they are identical only serves to glom more concepts into one, undermining the effort to understand component processes. While a critical interrogation of the relationship between WM and attention is essential to gain traction on these concepts, however, we should consider which routes for examining the relationship will generate testable predictions and theoretical progress.

## How should attention and working memory be defined and taxonomized?

Oberauer aptly points out that ‘attention’ is a widely used term with diverse meanings. One of the most fundamental properties of attention, however, is that it is capacity-limited and therefore must be selective. In that sense, the theoretical distinction between conceptions of attention as a *limited resource* vs. a *selection mechanism* may be flawed; instead, those may be inter-twined properties that jointly describe attention—and they describe WM too. By examining the conditions that influence selection, for WM and perceptual attention, we may better understand what limits them both.

While Oberauer indicates that there is broad consensus as to the meaning of WM, that consensus belies both the complexity of the function and the varied usages of the term. Like attention, WM comprises multiple concepts, abilities, and processes. It can refer to a distributed *system* for short-term retention, a *process* of short-term maintenance, or a *store* for temporary representations. WM can operate in any sensory modality (e.g., verbal or visual) and holds information at several levels: WM maintains abstract goals that shape entire episodes, context-specific task rules, and concrete sensory content that feeds into meeting those rules. Tests of WM place variable demands on perceptual encoding, updating or manipulation, feature binding, and interference resolution, as well as memory retrieval and decision-making when memory is probed. WM content can vary in its motivational relevance and in the goal(s) it is ultimately being applied toward. These many sub-processes, content domains, and intentional states can engage distinct brain regions and networks, which likely underlies the currently fractured perspectives about how WM operates (e.g., Christophel, Klink, Spitzer, Roelfsema, & Haynes, 2017; Constantinidis et al., 2018; Leavitt, Mendoza-Halliday, & Martinez-Trujillo, 2017; Lundqvist, Herman, & Miller, 2018; Scimeca, Kiyonaga, & D’Esposito, 2018; Xu, 2017). When we evaluate the relationship between the concepts of attention and working memory, therefore, we should recognize that the reciprocity between the two will depend on what processes and contents are at play.

## Under what circumstances should attention and working memory interact?

WM performance is often unimpaired by concurrent perceptual attentional demands (and vice versa), fueling a common sense argument against the idea that the two are interdependent. However, this argument assumes (1) that all attentional demands are equally demanding, (2) that they should compete for the same resource (regardless of the sub-processes engaged), and (3) that resource competition is always evident in behavior. Rather, two interleaved WM tasks may impair each other more than a WM task paired with a perceptual task (cf. Fougnie & Marois, 2006), not because they rely on distinct mechanisms, but because the demand inherent to WM is greater than when the object of attention is activated by perceptual input. Moreover, we would expect WM and perceptual attentional dual-task demands to impact each other more when they both load on the same attentional sub-processes (cf. Woodman & Chun, 2006). Finally, brain activity can reveal sensitivity to the interaction between WM and perceptual attention—even when behavior is unaffected—suggesting that control processes might sometimes compensate for the impacts of resource competition (e.g., Kiyonaga, Dowd, & Egner, 2017).

The interactions between WM and perceptual attention should also vary with their overlap in representational content (cf. Kim, Kim, & Chun, 2005). When perceptually attended stimuli are more similar to each other (e.g., in location or features) both neural and behavioral responses associated with individual items are degraded (Pelli & Tillman, 2008; Reddy, Kanwisher, & VanRullen, 2009). Likewise, visual WM is more disrupted by visual distraction from the same content category (Jha & Kiyonaga, 2010; Sreenivasan & Jha, 2007; Yoon, Curtis, & D’Esposito, 2006), and the interplay between WM and visual attentional demands is continuously graded with the similarity between them (Kiyonaga & Egner, 2016; Magnussen & Greenlee, 1992; Rademaker, Bloem, De Weerd, & Sack, 2015). Unrelated content may be trivial to segregate, therefore manifesting as absence of evidence for the interaction between WM and attention. To discover whether capacity constraints and selection mechanisms are truly distinct between these constructs, however, a fair comparison will account for the correspondence between the processes and content in each domain.

## Why characterize the relationship between attention and working memory?

Oberauer’s review underscores that there are many potential ways to catalogue and test the relationship between WM and attention. The most fruitful of these approaches may become clearer if we ask why it is important to examine that relationship in the first place. WM is more than a simple storage capacity—it is a ubiquitous contributor to many other cognitive processes and indices of success. Yet we understand very little about what determines its capacity. If we harness the rich history of attention research to illuminate (potentially) comparable WM functions, it may exponentially advance our understanding of WM. Following this approach, many studies have now shown that WM operates by the same principles that govern attentional selection, and that WM content can influence behavior as if it’s being attended in the environment (Johnson et al., 2013; Kiyonaga & Egner, 2014, 2016; Saad & Silvanto, 2013). The comparison of WM to attention in this manner therefore creates testable predictions that can advance theories of both concepts.

Oberauer lists pressing questions to guide future investigations. This work should probe (1) the *functional* role of attentionally modulated sensory cortical signals, (2) how mnemonic and perceptual sensory content may be processed simultaneously, and (3) how representations and maintenance mechanisms across several levels of the processing stream may be integrated to explain the capacity and control of WM and attention. In daily life, we constantly rely on WM to achieve our goals, and we suffer to varying degrees from the simultaneous stream of demands for our attention in the environment. Rather than simply reflect a shortcoming in our cognitive capacity, however, the susceptibilities of WM to other demands may provide a window into the structure and function of the system. By examining when interactions between WM and perceptual attentional demands are present or absent, we can zero in on the conditions that optimize human performance.
